# An innovative three-layer strategy in response to a quartan malaria outbreak among forest goers in Hainan Island, China: a retrospective study

**DOI:** 10.1186/s40249-022-01015-6

**Published:** 2022-09-14

**Authors:** Yuchun Li, Yingjuan Huang, Renqiang Chen, Weizhen Huang, Huanzhi Xu, Rongshen Ye, Shaoling Huang, Ji Zhen, Xiaodan Wen, Guoyi Wang, Yong Liu, Haishan Li, Zaichun Zheng, Jian Wang, Guoshen Wang, Chong Chen, Wen Zeng, Feng Meng, Xiaoming Huang, Guangze Wang, Bing Yang, Yan Chen

**Affiliations:** 1 Hainan Provincial Center for Disease Control and Prevention, No. 40 Haifu Road, Haikou, 570203 People’s Republic of China; 2 Sanya City’s Center for Disease Control and Prevention, No.674, Jiefang Third Road, Sanya, 570203 People’s Republic of China; 3 Wuzhishan City’s Center for Disease Control and Prevention, No. 26, Aoya Road, Wuzhishan, 572200 People’s Republic of China; 4 Baoting County’s Center for Disease Control and Prevention, No. 2 Wenquan South Road, Baoting County, 572300 People’s Republic of China; 5 Wanning City’s Center for Disease Control and Prevention, No.70 Guangming South Road, Wanning City, 571500 People’s Republic of China; 6 Dongfang City’s Center for Disease Control and Prevention, Intersection of Liberation West Road and Harvest Road, Dongfang City, 572600 People’s Republic of China; 7 Danzhou City’s Center for Disease Control and Prevention, No. 2000 Zhongxing Avenue, Danzhou City, 571700 People’s Republic of China; 8 Qiongzhong County’s Center for Disease Control and Prevention, Intersection of Baihua Road and Education Road, Qiongzhong County, 572900 People’s Republic of China; 9 Tunchang County’s Center for Disease Control and Prevention, No. 6 Jiefang Road, Tunchang County, 571600 People’s Republic of China; 10 Ledong County’s Center for Disease Control and Prevention, Nearby Secondary Health Vocational and Technical School, Ledong County, 572500 People’s Republic of China; 11 Changjiang County’s Center for Disease Control and Prevention, Intersection People North Road and Huimin Road, Changjiang County, 572700 People’s Republic of China; 12 Baisha County’s Center for Disease Control and Prevention, Weisheng Road, Baisha County, 572800 People’s Republic of China; 13 Qionghai County’s Center for Disease Control and Prevention, No. 17 Fuhaiheng South Road, Qionghai County, 571400 People’s Republic of China; 14 Lingshui County’s Institute of Health Supervision, Shuangyong Road, Lingshui County, 572400 People’s Republic of China

**Keywords:** Malaria, Three-layer strategy, Outbreak, Elimination, Hainan, China

## Abstract

**Background:**

An outbreak of *Plasmodium* *malariae* infection among forest goers in Sanya City of Hainan Island, China was reported in 2015. In response to this outbreak, an innovative three-layer strategy (TLS) targeted forest goers was adapted based on the 1-3-7 approach.

**Main text:**

Key elements of TLS are: (i) The village with five malaria cases and adjacent villages were set as the first layer. All residents including forest goers were taken as the high-risk population (HRP). Active case detection (ACD) by blood smear microscopy and PCR was selected as the primary measure, and passive case detection (PCD) as complementary measure. One case was identified under TLS implementation. (ii) The township with cases (Gaofeng Town) and the nearby towns were chosen as the second layer. Only forest goers were screened by ACD, while PCD as a routine screening method. 7831 blood smears collected by ACD and PCD and tested with negative results. (iii) The city with cases (Sanya City) and others 12 counties/county-level cities were selected as the third layer. Malaria cases were monitored passively. A total of 77,555 blood slides were screened by PCD with zero positive sample. For each layer, the malaria vector mosquitoes were monitored using light traps, cattle-baited/human-bait traps*.* *Anopheles* *minimus* (dominant species), *An.* *sinensis* and *An.* *dirus* were captured. Vector control measures mainly include insecticide residual spraying and long-lasting insecticide nets*.* The capacity of clinicians, public health practitioners and laboratory technicians has been improved through training. During 2016‒2018, TLS and chemoprophylaxis were implemented in the same areas. In the first layer, all residents were monitored by ACD, and malaria chemoprophylaxis were distributed, 89.5% of forest goers were using chemoprophylaxis against malaria. The blood smears (3126 by ACD plus 1516 by PCD) were with zero positive results. Chemoprophylaxis and ACD were offered to forest goers once a year, and PCD in residents as a complementary measure in the second and third layer, 77.8% and 95.1% of forest goers received chemoprophylaxis. In each layer, vector surveillance and control of malaria and trainings for medical staff were still in place.

**Conclusions:**

TLS was effective in blocking the outbreak by *P.* *malariae* among forest goers in Hainan in malaria elimination stage. However, whether it could prevent the malaria resurgence in the post-elimination phase needs to be further assessed.

**Graphical Abstract:**

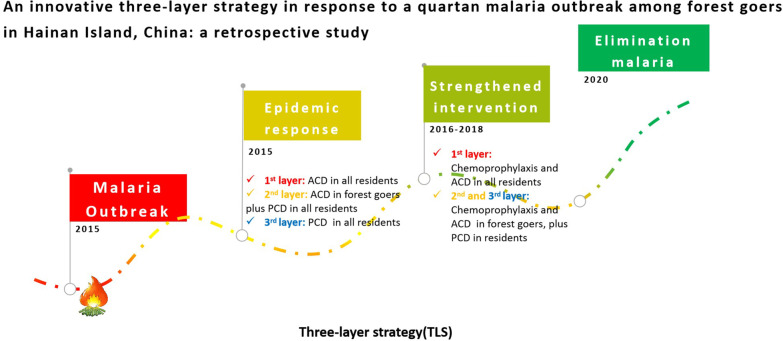

**Supplementary Information:**

The online version contains supplementary material available at 10.1186/s40249-022-01015-6.

## Background

Historically, malaria has been one of the most important infectious diseases in China. Hainan and Yunnan provinces were the main malaria transmission areas in the People's Republic of China [1, 2]. Prior to 2010, indigenous cases of falciparum malaria and vivax malaria were frequently detected in Hainan. Hainan is geographically characterized by mountains, hills, plateaus and plains, and the tropical monsoon and marine climates jointly produce a generally warm temperature not only for cultivating tropical plants (coconuts, areca nut and rubber trees, and more) but also for the breeding of *Anopheles* *dirus* and *An.* *minimus* [3]. Malaria cases in Hainan Island were mainly distributed in patches in the southwestern region of the island, and malaria infection in forest goers cannot be ignored in Hainan. Sanya City was one of malaria endemic (*Plasmodium* *vivax* and *P.* *falciparum*) cities in Hainan Island, which is located at the southern tip of Hainan Island [4, 5]. *An.* *sinensis* was considered to be a major vector in Sanya, *An.* *minimusis* was captured in mountainous regions at times.

Forest goers referred to residents and migrants who sleeping overnight in the mountains for a living by picking and planting. Forest goers were among the high-risk populations in Hainan since the 1990s [6]. In 2002, forest goers (30.9%, 95/307) were more than twice as likely to be infected than non-forest-goer residents (15.2%) [7]. In 1991, an investigation in Nanqiao of Wanning City showed that the infection rate of malaria among forest goers (49.4%, 118/239) was significantly higher than that among non-forest-goers (8%, 11/138) [8]. The factors related to malaria infection rate in forest goers including the frequency of staying in the mountains, whether to take antimalarial chemoprophylaxis, the acceptance of antimalarial propaganda, and mosquito control measures [9–11]. Hainan Island has been engaged in malaria control and elimination in forest goers since the 1990s. Been supported by the Global Fund to Fight AIDS, Tuberculosis and Malaria, the strategy for the prevention and control of malaria in forest goers  including mass drug administration (MDA) that focused on patients and the surrounding population (family members or co-workers), epidemiology investigations on patients, timely surveillance of vector dynamics, and vector control measures [such as insecticide residual spraying (IRS), insecticide-treated nets (ITNs) or long-lasting insecticidal nets (LLINs)], has been intensified since 2003 [12]. Seasonal anti-malaria measures were carried out uninterruptedly in spring and autumn annually in Hainan resulting in a decline of malaria incidence [13].

In 2010, Hainan joined the National Malaria Elimination Programme (NMEP). In Hainan Island there were eight Class I counties (endemic counties of *P.* *falciparum*) and ten Class II counties (endemic counties of *P.* *vivax*) [14]. Hainan Island officially launched malaria elimination in 2011. Subsequently, the 1-3-7 approach have been applied in the disposal of foci since 2012, which refer as following: case reporting within 1 day, case investigation within 3 days, and focus investigation and action within 7 days. Under the requirement of 1-3-7 approach, every reported case was confirmed by microscopy and PCR, and every focus file was collected and reported through the Parasitic Diseases Information Reporting Management System (PDIRMS). The last indigenous malaria case of *P.* *vivax* in Hainan was reported in Sanya in 2012.

From 2013, only imported malaria cases were reported in Hainan Island, and every imported malaria focus was classified and disposed according to the guidelines of the 1-3-7 approach. In 2015, there was an outbreak reported in Sanya, which was induced by indigenous cases infected by *P.* *malariae* among forest goers [15]. Based on the 1-3-7 approach, an innovative three-layer strategy (TLS) was designed and applied in the disposal of outbreak in 2015. From 2016 to 2018, the effectiveness of TLS was evaluated and mass drug administration by chemoprophylaxis were conducted in three layers. Hainan Province has achieved the goal of elimination malaria in 2019 and acquired the WHO certification of malaria elimination by field in 2021 [16]. This article summarizes the prevention and control measures of TLS strategy which administrated during the *P.* *malariae* malaria outbreak in 2015, and further outlines the lessons learned from the generation to evaluation from process.

### An outbreak of *P. malariae* malaria occurred in Sanya City, Hainan in 2015

**Fig. 1:**
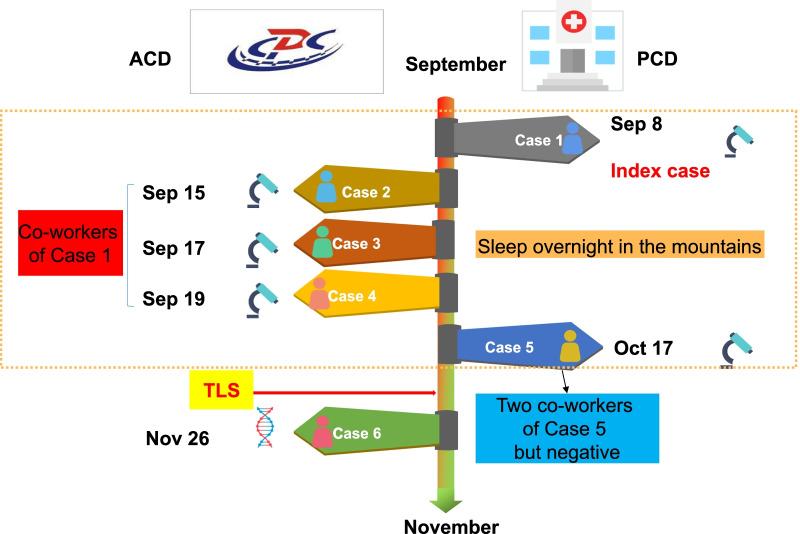
Information on the schedule of case exposure time and frequency, the method of detection, diagnosis and epidemiological characteristics in this outbreak. *ACD* active case detection, *PCD* passive case detection, *CDC* Center for Diseases Control and Prevention

The first malaria case was reported on September 7, 2015. A total of six indigenous *P.* *malariae* cases were sequentially detected by ACD and PCD surveillance. All of them were male farmers aged from 19 to 40 years. This outbreak was reported from three villages (Baolong, Zhanan, Lixin) of Gaofeng town in Sanya City, respectively. Four cases in September, one case each in October and November, and no more cases were detected by PCD after that (Fig. 1).

**Case 1** Male, 31 years, farmer, lived in Baolong village. On September 7, 2015, a male outpatient with chills, fever, headache, and limb weakness was diagnosed with *P.* *malariae* infection by blood smear microscopy in Sanya Hospital of Agricultural Reclamation (SYAR). The case subsequently confirmed by blood smear microscopy and PCR in Hainan Provincial Malaria Diagnosis Lab (HPMDL). Combined with the epidemiological history (without overnight in abroad and blood transfusion) and laboratory findings, he was determined as an indigenous case, and further classified as forest goer (Case 1). According to the information provided by Case 1, another three co-workers were confirmed as the new cases by blood smear microscopy and PCR (**Case 2:** Male, 19 years, farmer, lived in Lixin village; **Case 3:** Male, 27 years, farmer, lived in Lixin village; **Case 4:** Male, 31 years, farmer, lived in Lixin village). The four cases reported to stay overnight to collect bodhi fruit.

**Case 5** Male, 40 years, farmer, lived in Zhanan village. On October 17, 2015, a male outpatient with chills, fever, headache, and limb weakness was diagnosed with *P.* *malariae* infection by blood smear microscopy in Nandao Township Hospital. The case subsequently confirmed by blood smear microscopy and PCR in HPMDL. Considering the epidemiological history (without overnight in abroad and blood transfusion) and laboratory findings, he was also determined as an indigenous case and classified as a forest goer.

**Case 6** Male, 25 years, farmer, lived in Lixin village. On November 26, 2015, a symptomatic malaria carrier was found by PCR in HPMDL after TLS implemented. Subsequently, the case was confirmed by microscopy as *P.* *malariae* infection. However, Case 6 had no history of overnight sleeping in mountain, and classified as a victim in village.

All cases were sequentially transferred to SYAR and hospitalized to receive treatment with a standard regimen of oral chloroquine phosphate for 3 days (600 mg on 1st day, and then 300 mg once a day on the 2nd and 3rd days of therapy), plus primaquine diphosphate for 8 days (22.5 mg per day) to ensure therapeutic compliance.

### Design of TLS and its application in the 2015 outbreak

**Fig. 2:**
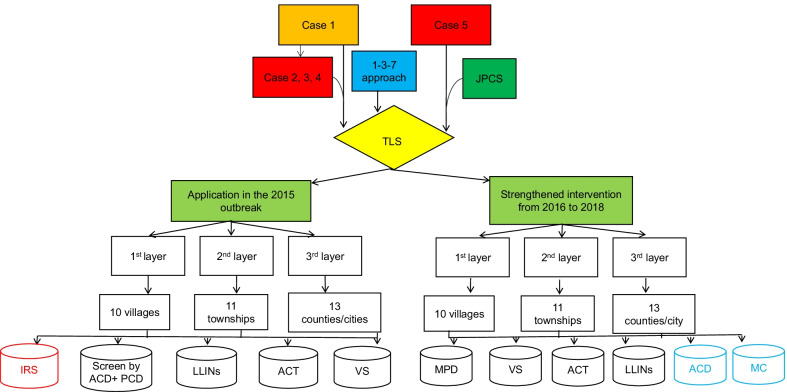
The skeleton map of three-layer strategy applied from 2015 to 2018 in Hainan. The red frame represents applied in the first layer only, but the blue frame represents various measures depend on different layers. *IRS* indoor residual spraying, *TLS* three-layer strategy, *LLINs* long lasting insecticide nets, *JPCS* joint prevention and control strategies, *ACD* active case detection, *PCD* passive case detection, *MPD* malaria parasite detection, *VS* vector surveillance, *ACT* attendees of capacity training, *MC* mass chemoprophylaxis

Based on the geographical distribution of five malaria cases (Case 1, 5 by PCD, and Case 2, 3, 4 by ACD), history of malaria joint defence and work urgency of elimination malaria, an innovative three-layer strategy (TLS, Fig. 2) was designed for expanded screening, PCD and ACD were optimally conducted as described in a previous study [17–19]. TLS was applied to prevent malaria transmission in the 2015 outbreak, more details showed as below, and the scope of three layers in details have showed in Additional files 1 and 3.

#### First layer

The villages with five malaria cases (Baolong, Zhanan and Lixin in Gaofeng Town, Sanya City) and adjacent villages, were regarded as the first layer. All residents who lived in the first layer were considered as the high-risk population (HRP). ACD screening was taken as primary measure in all residents by blood smear microscopy and SSU rRNA PCR assay [20], and PCD acted as complementary measure. During the implementation of  ACD, a total of 1774 slides were screened by blood smear microscopy and PCR. Thirteen positive samples were found by PCR. Confirmed by experienced microscopists from HPMDL, only Case 6 were identified and determined as indigenous cases of *P.* *malariae* infection (Table 1).

#### Second layer

The Gaofeng Town and ten adjacent towns, which involved to Yucai in Sanya city, Daan and Zhizhong in Ledong County, Xiangshui, Maogan, Nanlin, Sandao, Xinzhen in Baoting County and Changhao in Wuzhishan city, were chosen as the second layer. ACD screening for forest goers were conducted in ten towns. While the PCD as routine screening method was reinforced in the health establishments and diagnostic assays were carried out on all febrile patients. A total of 7831 blood slides were screened by PCD and ACD, and negative results were obtained (Table 1).

#### Third layer

Sanya City and others 12 counties/county-level cities were selected as the third layer, refered to Ledong, Baoting, Wuzhishan, Dongfang, Lingshui, Qiongzhong, Baisha, Changjiang, Wanning, Qionghai, Tunchang and Danzhou. Febrile forest goers were screened. The forest goers who with fever at the time or had a history of fever within the past one month were defined as HRP, PCD was the primarily routine measure for screening. A total of 77,555 blood slides were screened by PCD, plus 17,795 blood slides by ACD. All tests were negative (Table 1).

#### Malaria vector surveillance and control measures, medical staff training

Vector surveillance and control were also implemented as described previously [21, 22]. Anopheline mosquitoes were collected using light traps, cattle-baited/human-bait traps in every month throughout the year of 2015 and for at least three consecutive nights in a month. (i) *An.* *minimus* and *An.* *sinensis* (dominant species) were trapped in the first layer. (ii) In the second layer, *An.* *sinensis* was the dominant species. *An.* *minimus* and *An.* *dirus* were captured only in Wuzhishan City. (iii) In the third layer, *An.* *minimus* was captured in 4 counties (Changjiang, Danzhou, Baisha and Tunchang); and *An.* *dirus* was captured only in Baisha County, while *An.* *sinensis* was captured in every county except for Qionghai. The dynamics of vectors indicated that there were highly effective malaria vectors around the foci in Sanya City, and mosquito surveillance was necessary.

Vector control measures mainly include IRS for *An.* *sinensis* and *An.* *minimus,* and the distribution LLINs for *An.* *dirus* and *An.* *minimus.* (i) IRS with deltamethrin was only implemented in every focus in the first layer. (ii) A total of 6783 LLINs were distributed and covered in three layers involving 11 counties or cites except for Tunchang County and Wanning City (Table 2). In addition, the capacity of clinicians, public health personnel and laboratory personnel enhanced with the regular training at different service levels (Table 3; Additional file 2).

**Table 1:** Implementation of the three-layer strategy for epidemic response in 2015 outbreak and strengthened intervention from 2016 to 2018

Layer	No. of counties/cities	No. of towns	No. of villages	Epidemic response in 2015				Strengthened intervention from 2016 to 2018					
				PCD		ACD		MPD			ACD in forest goer	Chemoprophylaxis	
				No. of negative	No. of positive	No. of negative	No. of positive	2016	2017	2018		*n*	Coverage rate, %*
First layer	2	2	10	496	0	1770	1^a^	827	564	125	3126	2799	89.5
Second layer	4	11	–	6121	0	1710	0	6714	5788	5691	5118	3984	77.8
Third layer	13	–	–	77,555	0	17,795	0	99,586	176,943	97,630	10,364	9857	95.1
Total	–	–	–	84,172	0	21,275	1^a^	107,127	183,295	103,446	18,608	16,640	89.4

*ACD* active case detection, *PCD* passive case detection, *MPD* malaria parasite detection by microscopy, *TLS* three-layer strategy, – not applicable

^a^This data showed the number of cases after implementation of TLS, not including five cases which depending on 1-3-7 approach by ACD plus PCD

*Significant difference among three layers (Chi-square value = 979.6, *P* < 0.01)

**Table 2:** Malaria vector surveillance and control from 2015 to 2018 under the three-layer strategy

Layer	Vector surveillance (No. of mosquitoes collected)												Vector control^a^			
	2015			2016			2017			2018			No. of LLINs distributed			
	*An.* *minus*	*An.* *dirus*	*An.* *sinensis*	*An.* *minus*	*An.* *dirus*	*An.* *sinensis*	*An.* *minus*	*An.* *dirus*	*An.* *sinensis*	*An.* *minus*	*An.* *dirus*	*An.* *sinensis*	2015	2016	2017	2018
First layer	1	0	154	1	2	687	0	0	679	0	0	196	900	3348	598	490
Second layer	2	18	238	2	19	231	0	34	115	2	21	104	1047	1325	165	113
Third layer	274	19	3200	56	40	2713	22	3	3551	25	0	1272	6783	6003	1724	689
Total	277	37	3592	59	61	3631	22	37	4345	27	21	1572	8730	10,676	2487	1292

*LLINs* long-lasting insecticide nets

^a^Insecticide residual spraying (IRS) data were not shown, and IRS was implemented only in the address of six foci in Sanya

**Table 3:** Attendees of capacity training for response and intervention to malaria epidemic in 13 counties or cities of Sanya City, China from 2015 to 2018

Layer	No. of clinicians				No. of public health personnel				No. of laboratory technicians			
	2015	2016	2017	2018	2015	2016	2017	2018	2015	2016	2017	2018
First Layer	51	41	44	41	119	67	92	42	45	35	45	34
Second Layer	34	37	56	0	61	78	44	46	35	36	52	23
Third Layer	264	297	287	167	682	487	215	229	211	187	209	148
Total	349	375	387	208	862	632	351	317	291	258	306	205

### Strengthened intervention by TLS from 2016 to 2018

From 2016 to 2018, TLS was applied to prevent malaria re-establishment, and mass chemoprophylaxis (MC) was conducted in the three layers. The scope of strengthened intervention by TLS was the same as its in 2015. The chemoprophylaxis of administration by piperaquine phosphate, and doses for children decreased by weight or age. Villagers, including forest goers, were required to sign informed consent forms before administration of the drugs.

#### First layer

From March to June, and September in every year from 2016 to 2018, adult residents in the first layer were given a total dose of 600 mg per month. Chemoprophylaxis and ACD were given and implemented on all residents, including forest goers. 89.5% of forest goers accepted chemoprophylaxis. A total of 4642 blood smears (3126 by ACD plus 1516 by PCD) were diagnosed by microscopy (Table 1). No positive slides were obtained.

#### Second layer and third layer

In the second and third layer, 77.8% of 5489 forest goers received chemoprophylaxis in the second layer. Chemoprophylaxis and ACD were used once a year seeking for forest goers in the field. In the third layer, 10,364 people were classified as forest goers, and 95.1% of them received chemoprophylaxis (Table 1).

#### Malaria vector surveillance and control measures, medical staff training

Vector surveillance and control were implemented as described previously. During 2016–2018, (i) *An.* *minimus* and *An.* *sinensis* were also captured in the first layer, and *An.* *sinensis* was still the dominant species. LLIN distribution was considered as an effective measure for vector control in Hainan from 2016 to 2018 because of the existence of *An.* *dirus*. (ii) In the second and third laryer, *An.* *sinensis* was the dominant species. *An.* *minimus* and *An.* *dirus* were usually captured in all years from 2016 to 2018. LLINs and training were also distributed in the two layers, except for Tunchang County and Wanning City in the third layer. Training on clinicians, public health personnel and laboratory personnel was persisted from 2016 to 2018 for maintaining alertness of the general health services to suspected malaria.

## Discussion

Forest goers have the highest-risk of malaria infection in Hainan, mostly due to the abundant forest products and human behaviour. Abundant forest products, such as wood, honey, and wild animals, attracted the residents and mobile populations to work as forest goers [23]. The behaviour of staying overnight without using nets results in malaria infection. In the malaria control phase, an investigation in Nanqiao, Wanning City showed that overnight behaviour in mountains, the low usage rate of nets and the lack of malaria prevention knowledge were the key factors that affected the epidemics and control of malaria [24, 25]. In the elimination stage, the malaria outbreak in 2015 been mentioned above was caused by forest goers, and five of them had a history of staying overnight in mountains.

In the elimination phase outbreaks still happened in China, but was much less than control stage [26, 27]. The innovative TLS was adapted from 1-3-7 approach and first applied in the 2015 outbreak, and Case 6 was confirmed as an asymptomatic carrier. If 1-3-7 approach was implemented in this outbreak, only three cases associated with Case 1 and cases associated with Case 5 could be found. TLS enlarged the screening scope, improved the case detection ability and detected potential sources of infection, especially for the asymptomatic carriers and cases without treatment. In the outbreak of 2015, six cases were from different villages of the same township (Gaofeng). No malaria cases found outside Sanya City. It would alert us that different intervention measures need to tailor in different layers, which are like the prevention strategies against COVID-19 in China [28].

This outbreak was caused by *P.* *malariae* and more likely to be an imported-introduced case. The human-monkey mode of transmission is impossible because only 5 cases were found at this time. There have been no subsequent cases in the mountains, although forest goers have existed since the 1990s [6]. All of the malaria parasites in this outbreak were identified as *P.* *malariae,* not *P.* *simium,* which led to zoonosis in forest goers in Brazil[29]. Sanya is a tourist city with a large number of migrant people, including people from abroad and *An.* *minimus*, which is the main effective vector of malaria in Hainan, also exists in Sanya. The present study concluded that there was a high possibility of human-to-mosquito-to-human transmission in forest goers.

Which vector can transmit *P.* *malariae* remains a puzzle. There is no reports of *P.* *malariae* sporozoites being found in the salivary glands of vectors [30, 31]. In China, only Sanya has reported locally sequentially indigenous cases of *P. malariae* [32, 33]. Sporadic elderly cases of *P.* *malaria*, rather than young-to-middle aged groups in Sanya, have been reported in Guangdong and Shanghai, and no successive cases have been reported, although *An.* *sinensis* exists [30, 31]. At present, *An.* *sinensis* is widely distributed and the dominant species in China in recent years, while *An.* *minimus* is only found in Yunnan and Hainan. We presumed that *An.* *minimus* is more likely to transmit *P.* *malaria* in Sanya than *An.* *sinensis*. The transmission of *P.* *malaria* by *An.* *minimus* is currently a conjecture.

The cities or counties in the central part of Hainan Island, where *An.* *dirus* and *An.* *minimus* exists, are actively developing tourism resources for economic development [34], where the challenges in the control and prevention of imported malaria, forest goers remain as a high-risk group.

TLS was firstly applied in the 2015 outbreak, but there was no more similar scenarios in China can be used to explore scopes of the TLS for practicability after 2015. In addition, although there were at least two transmission chains in the perspective of epidemiological investigation, the relationship between two transmission chains is not certain and further investigation is urgently need.

## Conclusions

The innovative TLS was effective in blocking the outbreak by *P.* *malariae* among forest goers in Hainan at malaria elimination stage. However, it still need to be tailored to apply in malaria control or elimination in similar settings for outbreak disposal. Moreover, whether it could prevent re-establishment by the potential malaria in the post-elimination phase needs to be further assessed.

## Supplementary Information

**Additional file 1**: The areas of three layers in details as a part of TLS in 2015

**Additional file 2**: 1. Vector surveillance and control from 2015 to 2018 when implementation of the three-layer strategy. 2. Training in details in 13 counties or cities for response and intervention from 2015 to 2018

**Additional file 3**: TLS applied in the disposal of the outbreak in 2015, and in strengthening epidemic measures from 2016 to 2018, respectively. (A) Bodhi fruit; (B) ACD of malaria in 2015; and (C) MDA to prevent malaria from 2016 to 2018

## Data Availability

Not applicable.

## Abbreviations

CDC: Center for Diseases Control and Prevention
IRS: Indoor residual spraying
MDA: Mass drug administration
TLS: Three-Layer Strategy
LLINs: Long-lasting insecticide nets
FG: Forest goer
NMEP: National Malaria Elimination Programme
JPCS: Joint prevention and control strategies
ACD: Active case detection
PCD: Passive case detection
PDIRMS: Parasitic Diseases Information Reporting Management System
MPD: Malaria parasite detection
SYAR: Sanya Hospital of Agricultural Reclamation
HPMDL: Hainan Provincial Malaria Diagnosis Lab
HRP: High-risk population
ITNs: Insecticide-treated nets
ACT: Attendees of capacity training
MC: Mass chemoprophylaxis
