# Supplementary material for: An innovative three-layer strategy in response to a quartan malaria outbreak among forest goers in Hainan Island, China: a retrospective study
Source: Infect Dis Poverty. 2022 Sep 14;11:97. doi: 10.1186/s40249-022-01015-6 (PMC9473465; doi:10.1186/s40249-022-01015-6)
Supplement: Supplementary file 1 — Additional file 1: The areas of three layers in details as a part of TLS in 2015 [file 40249_2022_1015_MOESM1_ESM.docx]

Additional file 1 The areas of three layers in details as a part of TLS in 2015

| Layers | Names of counties/cities | Names of township | Names of village |
| --- | --- | --- | --- |
| 1^st^ Layer | Sanya | Gaofeng | Baolong† |
|  |  |  | Lixin† |
|  |  |  | Zhanan† |
|  | Baoting | Xinzheng | Shirang |
|  |  |  | Maowen |
|  |  |  | Xinzheng |
|  |  |  | Maopeng |
|  |  |  | Baodao |
|  |  |  | Shenna |
|  |  |  | Nangai |
| 2^nd^ Layer | Sanya | Gaofeng* | - |
|  |  | Yucai | - |
|  | Ledong | Daan | - |
|  |  | Zhizhong | - |
|  |  | Baoguo | - |
|  | Baoting | Xiangshui | - |
|  |  | Maogan | - |
|  |  | Nanlin | - |
|  |  | Xinzheng* | - |
|  |  | Sandao | - |
|  | Wuzhishan | Changhao | - |

| Layers | Names of counties/cities | Names of township | Names of village |
| --- | --- | --- | --- |
| 3^rd^ Layer | Sanya* | - | - |
|  | Ledong | - | - |
|  | Baoting* | - | - |
|  | Wuzhishan | - | - |
|  | Dongfang | - | - |
|  | Lingshui | - | - |
|  | Qiongzhong | - | - |
|  | Baisha | - | - |
|  | Changjiang | - | - |
|  | Waning | - | - |
|  | Qionghai | - | - |
|  | Tunchang | - | - |
|  | Danzhou | - | - |

†Baolong village consists of 4 units, Hongqi, Zhatao, Zhaban, Xianjin; Zhanan village consists of 8 units, Nanjin, Hongxing, Jiayue, Xincun, Baotu, Ganyou, Lingqu; Lixin village consists of 4 units, Zhayun, Zhaka, Zhaye, Zhachu, Zhaye, Xincun. * The data are shown in the previous layer. Little dash was not applicable.
