# Supplementary material for: An innovative three-layer strategy in response to a quartan malaria outbreak among forest goers in Hainan Island, China: a retrospective study
Source: Infect Dis Poverty. 2022 Sep 14;11:97. doi: 10.1186/s40249-022-01015-6 (PMC9473465; doi:10.1186/s40249-022-01015-6)
Supplement: Supplementary file 2 — Additional file 2: 1. Vector surveillance and control from 2015 to 2018 when implementation of the three-layer strategy. 2. Training in details in 13 counties or cities for response and intervention from 2015 to 2018 [file 40249_2022_1015_MOESM2_ESM.docx]

Additional file 2-1 Vector surveillance and control from 2015 to 2018 when implementation of the three-layer strategy

| Layer | Names of counties/cities | Vector surveillance | | | | | | | | | | | | Vector control* | | | |
| --- | --- | --- | --- | --- | --- | --- | --- | --- | --- | --- | --- | --- | --- | --- | --- | --- | --- |
|  |  | 2015 | | | 2016 | | | 2017 | | | 2018 | | | Distribution LLINs | | | |
|  |  | *An.*  *minus* | *An.*  *dirus* | *An. sinensis* | *An.*  *minus* | *An.*  *dirus* | *An.*  *sinensis* | *An.*  *minus* | *An.*  *dirus* | *An. sinensis* | *An.*  *minus* | *An.*  *dirus* | *An. sinensis* | 2015 | 2016 | 2017 | 2018 |
| First Layer | Sanya | 1 | 0 | 154 | 1 | 2 | 368 | 0 | 0 | 548 | 0 | 0 | 187 | 300 | 1,648 | 598 | 490 |
|  | Baoting | 0 | 0 | 0 | 0 | 0 | 319 | 0 | 0 | 131 | 0 | 0 | 9 | 600 | 1,700 | 0 | 0 |
| Second Layer | Ledong | 0 | 0 | 164 | 0 | 0 | 182 | 0 | 0 | 105 | 0 | 0 | 83 | 447 | 325 | 165 | 113 |
|  | Wuzhishan | 2 | 18 | 74 | 2 | 19 | 49 | 0 | 34 | 10 | 2 | 21 | 21 | 600 | 1,000 | 0 | 0 |
| Third Layer | Dongfang | 0 | 0 | 92 | 0 | 0 | 11 | 0 | 0 | 181 | 0 | 0 | 207 | 700 | 500 | 0 | 0 |
|  | Lingshui | 0 | 0 | 95 | 0 | 0 | 241 | 0 | 0 | 236 | 0 | 0 | 88 | 409 | 670 | 0 | 495 |
|  | Qiongzhong | 0 | 0 | 357 | 0 | 0 | 119 | 0 | 0 | 61 | 0 | 0 | 36 | 600 | 700 | 0 | 0 |
|  | Baisha | 3 | 19 | 910 | 9 | 0 | 242 | 0 | 0 | 418 | 1 | 0 | 224 | 600 | 700 | 0 | 0 |
|  | Changjiang | 100 | 0 | 327 | 0 | 40 | 116 | 1 | 3 | 93 | 1 | 0 | 9 | 1,564 | 424 | 309 | 134 |
|  | Wanning | 0 | 0 | 97 | 0 | 0 | 116 | 0 | 0 | 94 | 0 | 0 | 65 | 0 | 0 | 0 | 0 |
|  | Qionghai | 0 | 0 | 0 | 1 | 0 | 663 | 1 | 0 | 107 | 23 | 0 | 53 | 2,610 | 2,409 | 1,415 | 0 |
|  | Tunchang | 17 | 0 | 1,249 | 9 | 0 | 1,081 | 19 | 0 | 2,253 | 0 | 0 | 470 | 0 | 300 | 0 | 0 |
|  | Danzhou | 154 | 0 | 73 | 37 | 0 | 124 | 1 | 0 | 108 | 0 | 0 | 120 | 300 | 300 | 0 | 60 |
| Total | | 277 | 37 | 3,592 | 59 | 61 | 3,631 | 22 | 37 | 4,345 | 27 | 21 | 1572 | 8,730 | 10,676 | 2,487 | 1,292 |

**Additional file 2-2** Training in details in 13 counties or cities for response and intervention from 2015 to 2018

| Layer | Names of counties /cities | Clinician | | | | Public health doctor | | | | Laboratory technician | | | |
| --- | --- | --- | --- | --- | --- | --- | --- | --- | --- | --- | --- | --- | --- |
|  |  | 2015 | 2016 | 2017 | 2018 | 2015 | 2016 | 2017 | 2018 | 2015 | 2016 | 2017 | 2018 |
| First layer | Sanya | 23 | 18 | 20 | 24 | 36 | 24 | 20 | 22 | 29 | 18 | 26 | 19 |
|  | Baoting | 28 | 23 | 24 | 17 | 83 | 43 | 72 | 20 | 16 | 17 | 19 | 15 |
| Second layer | Ledong | 25 | 26 | 46 | 0 | 51 | 68 | 34 | 23 | 25 | 26 | 46 | 23 |
|  | Wuzhishan | 9 | 11 | 10 | 0 | 10 | 10 | 10 | 23 | 10 | 10 | 6 | 0 |
|  | Dongfang | 24 | 21 | 22 | 27 | 42 | 42 | 22 | 27 | 23 | 21 | 23 | 22 |
|  | Lingshui | 21 | 46 | 56 | 22 | 45 | 66 | 56 | 56 | 42 | 25 | 22 | 22 |
| Third layer | Qiongzhong | 105 | 0 | 25 | 24 | 0 | 0 | 0 | 0 | 2 | 0 | 27 | 4 |
|  | Baisha | 26 | 85 | 24 | 0 | 42 | 54 | 0 | 0 | 31 | 38 | 26 | 0 |
|  | Changjiang | 10 | 16 | 27 | 0 | 21 | 21 | 30 | 33 | 14 | 25 | 30 | 25 |
|  | Waning | 15 | 100 | 24 | 23 | 335 | 190 | 24 | 24 | 0 | 32 | 22 | 20 |
|  | Qionghai | 10 | 0 | 73 | 27 | 70 | 60 | 51 | 21 | 34 | 0 | 20 | 17 |
|  | Tunchang | 25 | 11 | 19 | 44 | 21 | 20 | 15 | 45 | 18 | 14 | 22 | 38 |
|  | Danzhou | 28 | 18 | 17 | 0 | 106 | 34 | 17 | 23 | 47 | 32 | 17 | 0 |
| Total | | 349 | 375 | 387 | 208 | 862 | 632 | 351 | 317 | 291 | 258 | 306 | 205 |

* The data are shown in the previous layer. Little dash was not applicable.
