# Supplementary material for: An innovative three-layer strategy in response to a quartan malaria outbreak among forest goers in Hainan Island, China: a retrospective study
Source: Infect Dis Poverty. 2022 Sep 14;11:97. doi: 10.1186/s40249-022-01015-6 (PMC9473465; doi:10.1186/s40249-022-01015-6)

**Additional file 3** TLS applied in the disposal of the outbreak in 2015, and in strengthening epidemic measures from 2016 to 2018, respectively.

(A) Bodhi fruit; (B) ACD of malaria in 2015; and (C) MDA to prevent malaria from 2016 to 2018.


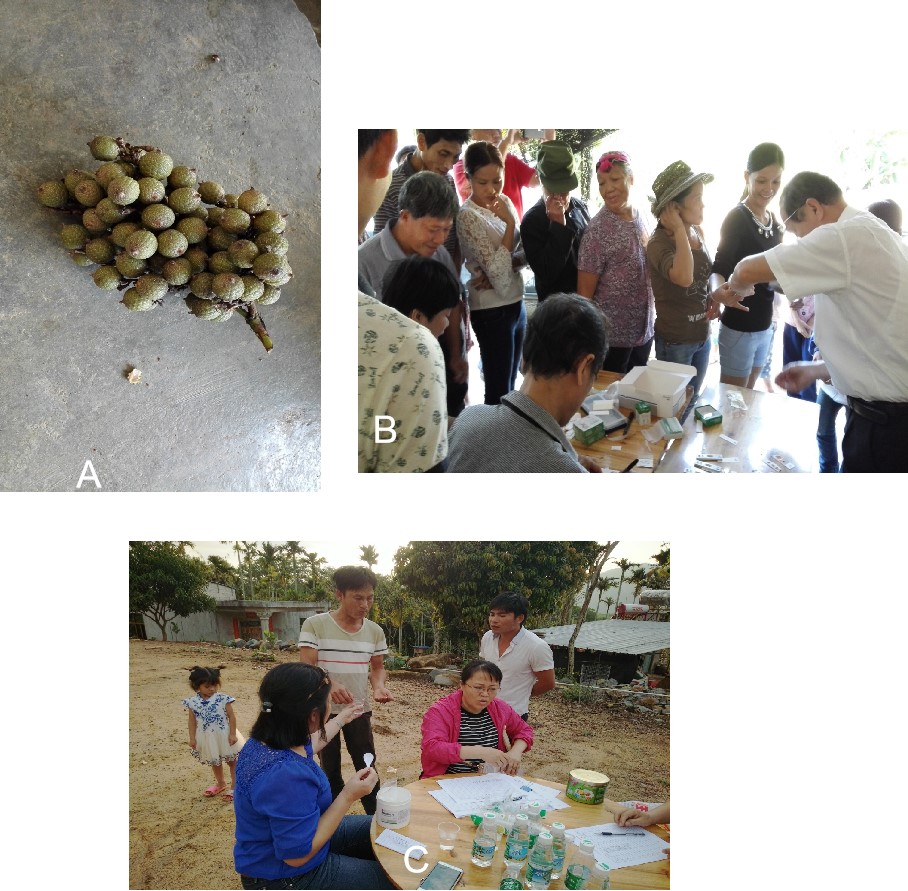

Supplement: Supplementary file 3 — Additional file 3: TLS applied in the disposal of the outbreak in 2015, and in strengthening epidemic measures from 2016 to 2018, respectively. (A) Bodhi fruit; (B) ACD of malaria in 2015; and (C) MDA to prevent malaria from 2016 to 2018 [file 40249_2022_1015_MOESM3_ESM.docx]
